# CT-Based Characterization of Fracture Patterns in Pediatric Lumbar Spondylolysis

**DOI:** 10.2106/JBJS.OA.25.00272

**Published:** 2025-11-25

**Authors:** Vivien Chan, Robert G. Watkins, Kenneth D. Illingworth, Corey T. Walker, David L. Skaggs

**Affiliations:** 1 Cedars Sinai Spine, Cedars Sinai Medical Center, Los Angeles, California; 2 Section of Neurosurgery, University of Calgary, Calgary, Alberta, Canada

## Abstract

**Background:**

Spondylolysis is a stress fracture of the pars interarticularis. Precise fracture morphology may help dictate management and dictate whether direct repair with an intralaminar screw is possible. The aim of this study was to characterize the specific fracture patterns of the pars interarticularis in pediatric patients with spondylolysis.

**Methods:**

This was a single-center retrospective cohort study. Patients were included if they were aged younger than 21 years with lumbar spondylolysis or Grade 1 isthmic spondylolisthesis on computed tomography (CT) imaging. On sagittal CT, total pars length (mm), inferior edge of inferior articular process to spondylolysis (mm), superior edge to spondylolysis (mm), size of spondylolysis gap (mm), and width of pars (mm) at fracture site were measured. The angle of the pars fractures were characterized in reference to the long axis of the pars.

**Results:**

There were 32 patients with 59 total spondylolyses included in this study. The mean age was 15.1 ± 1.9 years, and 44% (n = 14) were female. 7 (22%) patients had grade 1 spondylolisthesis. There were 15 fractures (25%) that had less than 0.5-mm gap, and the remaining 44 spondylolysis had a mean gap size of 2.4 ± 1.3 mm (range 0.9-6.4 mm). The mean total pars length was 37.9 ± 3.7 mm (range 31.6-45.8 mm). The mean measurement from the superior edge to the spondylolysis site was 12.1 ± 2.9 mm (range 6.1-18.8 mm). The average percentage of superior edge to spondylolysis/total pars length was 32.0% (range 17%-52%). Pars fracture angles ranged from 77° to 165° to the long axis of the pars. A majority (n = 40, 68%) of the pars fractures were between 110 and 140°. There were 12 fractures (20%) that were <110° and 7 fractures (12%) that were >140°.

**Conclusions:**

Pars fractures typically occur approximately one-third of the distance from the superior edge of the pars. However, considerable variability exists in their exact location along the pars. A clearer understanding of these fracture patterns may help refine surgical techniques and improve surgical outcomes for this patient population.

**Level of Evidence:**

Level III. See Instructions for Authors for a complete description of levels of evidence.

## Introduction

Spondylolysis is a defect or stress fracture of the pars interarticularis. The overall prevalence in the pediatric population is 3% to 6%, with increasing prevalence with increasing age^1^. Pars fractures are often associated with repetitive lumbar hyperextension and rotation, making it particularly prevalent among young athletes engaged in activities such as gymnastics, soccer, baseball, and dance^2,3^. While many cases are asymptomatic or resolve with conservative treatment, in some patients the symptoms persist or progress necessitating surgical intervention^4^. Surgery is indicated for patients who have failed conservative management with pain persisting for more than 6 months. One surgical option is a direct pars repair using an intralaminar screw. Contraindications for this procedure include disk degeneration at the pathological level, facet degeneration at the pathological level, and spondylolisthesis greater than Meyerding grade 1.

Understanding the detailed morphology of pars fractures is critical for optimizing direct pars repairs. Anatomic factors such as where the fracture occurs along the pars, orientation of the fracture, and width of the pars can influence the ability to place an intralaminar screw and subsequent bony fusion after intralaminar screw fixation^5^. However, despite its clinical relevance, the specific morphologic patterns of pars fractures in the pediatric population remain poorly characterized. The objective of this study was to characterize fracture patterns of the pars interarticularis and pars morphology in pediatric patients with spondylolysis. By identifying common morphologic patterns, we hope to better understand this pathology and optimize and improve surgical approaches for managing pediatric spondylolysis.

## Methods

### Study Design and Data

This was a single-center retrospective study. We included patients at the age of 20 years and younger with a lumbar pars fracture diagnosed on computed tomography (CT) imaging from February 2022 to October 2024. We included patients with spondylolysis and Meyerding grade 1 isthmic spondylolisthesis that are amendable for direct pars repair. We excluded patients who had prior spine surgery, a concurrent diagnosis of scoliosis, and isthmic spondylolisthesis with Meyerding grade higher than 1. Patient demographics were abstracted from electronic medical records. Race and ethnicity were not abstracted for this radiological study. Electronic imaging data were used to determine pars fracture characteristics, including level of fracture, side, and presence of spondylolisthesis. The study protocol was reviewed and approved by the institutional review board (IRB).

### Measurements and Statistical Analysis

**Fig. 1:**
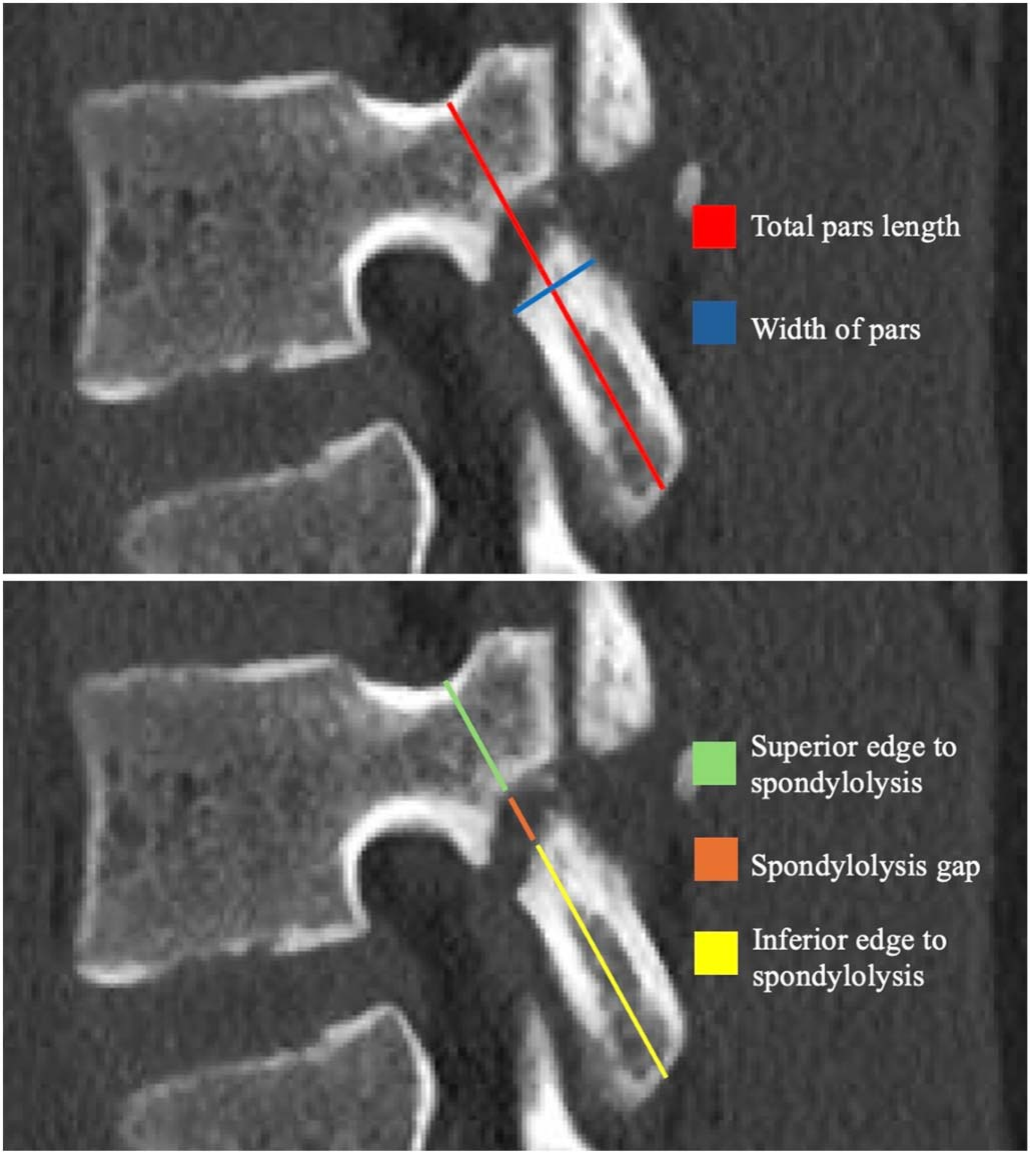
Pars measurements.

Measurements of interest were performed using the sagittal sequence of the lumbar CT (Fig. 1). Measurements included (1) total length of pars defined as length from where the pedicle and superior articular process meets to the inferior edge of the inferior articular process (mm), (2) distance from where the pedicle and superior articular process meets (superior edge) to the fracture (mm), and (3) distance from the inferior edge of the inferior articular process (inferior edge) to the fracture (mm), width of the pars adjacent to the fracture (mm), and size of the spondylolysis gap (mm). The superior edge to the spondylolysis over total length was calculated as a percentage. Using the long axis of the pars as reference, the angle of the pars fractures were characterized in degrees, with 90° being perpendicular to the long axis of the pars. Descriptive statistics were used for analysis. Discrete variables were reported as proportions. Continuous variables were reported as mean with standard deviation.

## Ethical Approval Statement

This study was approved by the local IRB.

## Results

### Patient Characteristics

There were 32 patients with 59 total pars fractures included in this study (Table I). The mean age was 15.1 ± 1.9 years. There were 44% (n = 14) female patients. Of the 32 patients, 78% (n = 25) had spondylolysis and 22% (n = 7) had Grade 1 spondylolisthesis. There were 25 bilateral pars fractures and 9 unilateral pars fractures (Left: 3, Right: 6). Of the 59 pars fractures, 48% (n = 28) were on the left side and 53% (n = 31) were on the right side. The most common level was L5 (54%, n = 32), followed by L4 (29%, n = 17). There were 2 pars fractures (3%) at L2 and 8 pars fractures (14%) at L3.

**TABLE I:** Patient Demographics

	N = 32, 59 Spondylolyses
Age (mean ± SD)	15.1 ± 1.9 yrs
Female (n, %)	14/32 (44)
Level of spondylolysis (n, %)	
L2	2/59 (3)
L3	8/59 (14)
L4	17/59 (29)
L5	32/59 (54)
Left side (n, %)	28 (48)
Spondylolysis or grade 1 spondylolisthesis (%)	
Spondylolysis	25/32 (78)
Grade 1 spondylolisthesis	7/32 (22)

### Pars and Pars Fracture Morphology

Pars measurements are summarized in Table II. Of the 59 pars fractures, 15 (25%) had a fracture gap of less than 0.5 mm. The remaining 44 pars fractures had a mean gap size of 2.4 ± 1.3 mm (range 0.9-6.4 mm). In patients with spondylolysis without spondylolisthesis, the mean gap size was 1.5 mm ± 1.3 mm (range 0.0-4.6 mm). Patients with grade 1 spondylolisthesis had a mean gap size of 3.1 mm ± 1.8 mm (range 1.0-6.4 mm). The mean total pars length was 37.9 ± 3.7 mm. The range of total pars length was 31.6 mm to 45.8 mm. The mean width of the pars on sagittal CT adjacent to the pars fracture was 8.2 ± 1.5 mm (range 5-10.7 mm).

**TABLE II:** Pars Measurements

	Mean ± SD	Range
Spondylolysis gap	2.4 ± 1.3 mm	0.9-6.4 mm
Total pars length	37.9 ± 3.7 mm	31.6-45.8 mm
Superior edge of pars to spondylolysis	12.1 ± 2.9 mm	6.1-18.8 mm
Inferior edge of pars to spondylolysis	24.0 ± 3.3 mm	15.9-35.1 mm
Width of pars at spondylolysis	8.2 ± 1.5 mm	5-10.7 mm
Angle of the fracture relative to long axis of pars	123° ± 18°	77°-165°

**Fig. 2:**
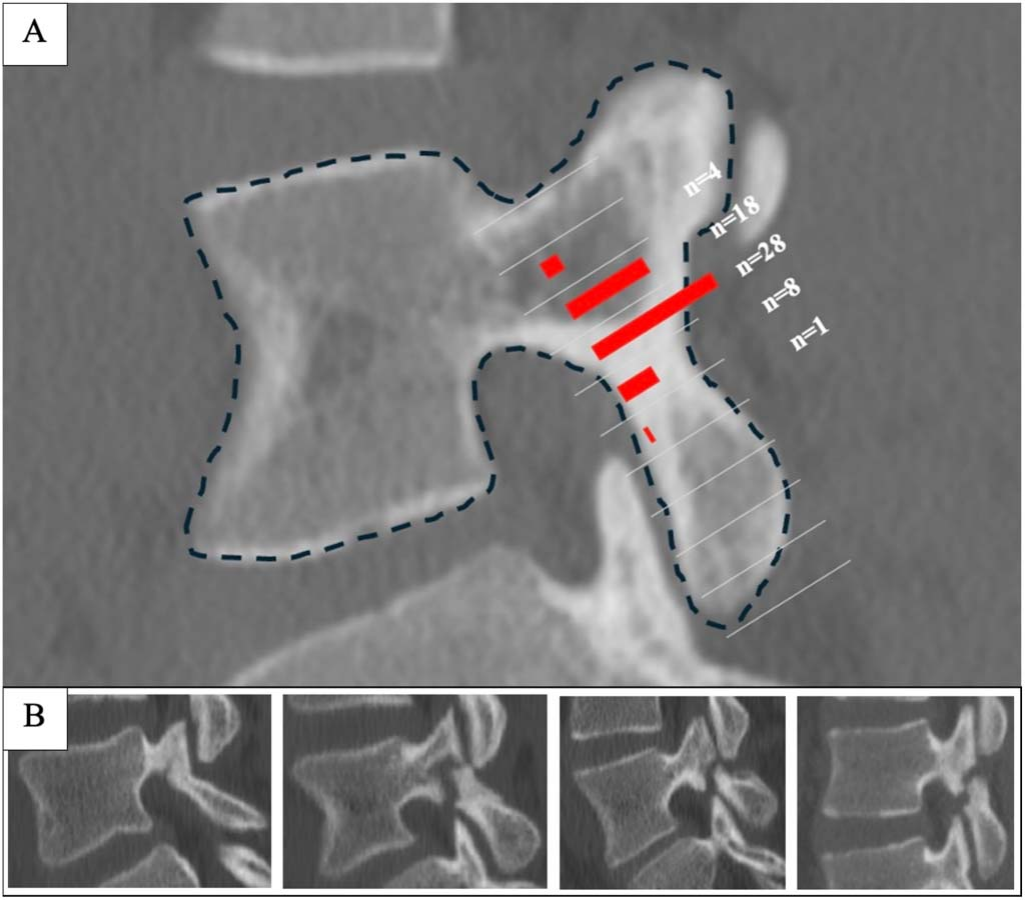
**Fig. 2-A** Location of pars fracture site along the pars interarticularis. **Fig. 2-B** Examples of variability in location of pars fracture along the pars interarticularis.

The mean measurement from the inferior edge to the spondylolysis site was 24.0 ± 3.3 mm [range 15.9-35.1 mm]. The mean measurement from the superior edge to the spondylolysis site was 12.1 ± 2.9 mm [range 6.1-18.8 mm]. The average percentage of superior edge to spondylolysis/total pars length was 32% [range 17%-52%]. The most common location of the fracture was 30 to 40% from the superior edge, observed in 48% of cases (n = 28) (Fig. 2). In 31% of cases (n = 18), the fracture occurred 20 to 30% from the super edge. The fracture occurred 40% to 50% from the superior edge in 14% of cases (n = 8). The fracture occurred 10% to 20% from the superior edge in 7% of cases (n = 4). There was 1 patient (2%) with a fracture 50% to 60% from the superior edge.

**Fig. 3:**
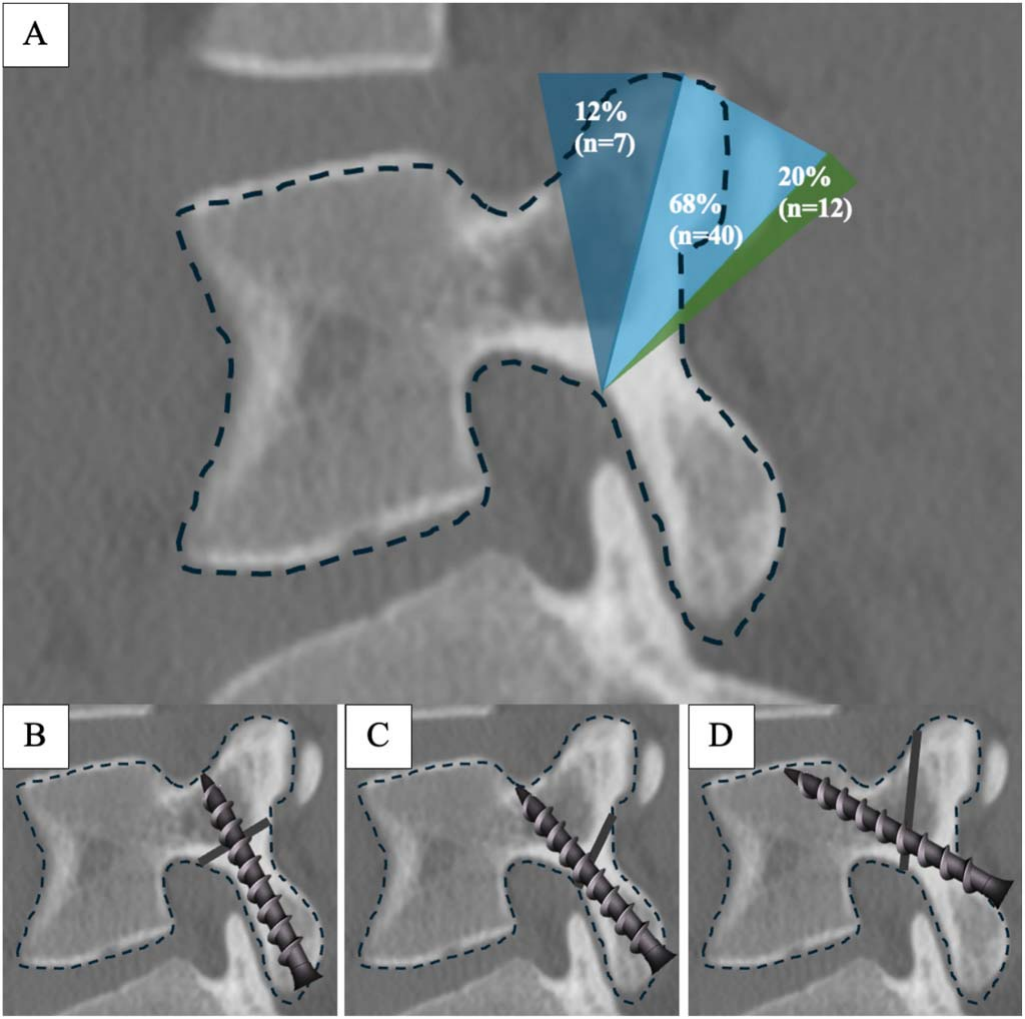
**Fig. 3-A** Angle of pars fracture relative to the long axis of the pars interarticularis: <110° = 12%, 110 to 140° = 68%, and >140° = 20%. Ideal intralaminar screw trajectory for (**Fig. 3-B**) perpendicular, (**Fig. 3-C**) oblique, and (**Fig. 3-D**) acute fractures.

Pars fracture angles ranged from 77° to 165° to the long axis of the pars. The mean was 123° ± 17.6° (Table II). A majority (n = 40, 68%) of the pars fractures were between 110 and 140° (Fig. 3). There were 12 fractures (20%) that were <110° and 7 (12%) fractures that were >140° (Fig. 3).

## Discussion

This was a retrospective cohort study that aimed to characterize pars fracture patterns and pars morphology in pediatric patients with spondylolysis. Our findings show that pars fractures most commonly occur approximately one-third of the distance from the superior edge of where the pedicle meets the superior articular process, with nearly half of fractures (48%) occurring between 30% and 40% of the total pars length from the superior edge. We identified 3 main patterns of fracture orientation: (1) perpendicular (<110°), (2) oblique (110-140°), and (3) acute angulation (<140°). The majority of fractures (68%) had angles between 110° and 140° relative to the long axis of the pars. In all our measurements for pars and fracture morphology, there was considerable variability. To our knowledge, this was the first study characterizing fracture patterns and pars morphology in pediatric patients with spondylolysis.

Although no prior studies have specifically characterized fracture patterns in pediatric spondylolysis, several investigations have examined morphological factors associated with its development. L5 is the most common level affected by spondylolysis. Masharawi et al. found that the change in orientation from sagittal articular facets at L1 to frontally oriented facets at L5 was significantly greater in individuals with spondylolysis, when compared with a control group^6^. Compared with the control group, patients with spondylolysis had vertebral bodies that were shorter posteriorly, more lordotic, longer lamina, longer isthmus, and longer and wider vertebral canal^7^. Hollenberg et al. proposed a classification system for grading lumbar spondylolysis to distinguish between stress reaction, active spondylolysis, and inactive spondylolysis^8^. In this classification system, stress reactions (Grade 1) were characterized by T2 signal abnormality of the pars without a pars fracture identified^8^. Grade 2 was defined as having T2 signal abnormalities with thinning, fragmentation, or irregularity of the pars interarticularis^8^. Grade 3 was defined as having complete unilateral or bilateral spondylolysis with T2 signal. Chronic pars fractures were defined as complete spondylolysis without T2 signal^8^. Hollenberg et al. proposed a grading system to classify lumbar spondylolysis based on magnetic resonance imaging (MRI) findings. In this system, Grade 1 represents a stress reaction characterized by T2 signal abnormalities without visible fracture; Grade 2 includes T2 signal with thinning, fragmentation, or irregularity of the pars; and Grade 3 denotes complete unilateral or bilateral spondylolysis with persistent T2 signal. Chronic fractures are defined as complete defects without T2 signal^8^. Although these foundational studies highlighted the importance of understanding variations in spondylolysis and how these variations may affect outcomes, precise classification of the pars fracture patterns is needed to guide and improve surgical approaches.

Buck repair is a surgical technique used to directly treat lumbar spondylolysis by placing an intralaminar screw across the pars fracture, with or without autologous bone grafting^9^. Previous studies have reported that this technique is both safe and effective^10^. A meta-analysis by Mohammed et al. reported pooled rates of 84% for both bony fusion and favorable clinical outcomes^5^. However, the overall pooled complication rate was 13%, with reported complications including screw breakage, screw loosening, and sciatica requiring revision surgery^5^. In recent years, there has been renewed interest in Buck repair due to the adoption of enabling technologies that improve screw placement accuracy and allow for minimally invasive approaches^11^. The typical screw trajectory is medial-to-lateral and caudal-to-cranial, following the long axis of the pars. Bone grafting options include decorticating and grafting either the dorsal pars^12^ or the lateral pars^13^. Findings from our study may help guide the application of Buck repair, which requires precise intralaminar screw placement within a narrow corridor to ensure optimal bony purchase. In our cohort, 25% of fractures were hairline, with gaps measuring less than 0.5 mm. The remaining 75% had gaps greater than 0.5 mm, with a mean gap of 2.4 ± 1.3 mm (range: 0.9-6.4 mm). Fractures with larger gaps may benefit from bone grafting or techniques that close the fracture gap, such as using a lag screw in Buck repair or alternative constructs such as pedicle-rod-hook or screw-rod systems^11,14-16^.

The mean superior edge-to-fracture distance was 12.1 mm, corresponding to 32% of total pars length. Nearly half of the pars fractures in our study occurred between 30% and 40% of the total pars length from the superior edge. This suggests a preferential fracture zone that may correspond to increased mechanical stress during lumbar extension and loading. Previous studies have reported a more horizontal sacrum leads to higher stress on the L5 pars interarticularis by both the L4 inferior articular facet superiorly and S1 superior articular facet inferiorly^17^. Similarly, Finkel et al. found patients with L5 spondylolysis have significantly more horizontal L5 pars that is closer to both the L4 inferior articular process and S1 superior articular process^18^. Fractures located within the upper third of the pars may be more amenable to lateral pars grafting, as the fracture line can lie ventral to the superior facet complex—necessitating resection of the inferior articular process of the level above to expose the dorsal aspect of the defect. However, fractures closer to the middle of the pars are good candidates for dorsal bone grafting.

Dunn et al. found that all incomplete pars fractures involved a break in the inferomedial cortex of the pars, while the superior cortex remained intact—suggesting that these fractures propagate in an inferior-to-superior direction^19^. Fractures may initiate inferiorly, with variation in their extension toward the superior cortex. In our study, we observed a wide range of fracture angles relative to the long axis of the pars, ranging from 77° to 165°. Three characteristic patterns emerged. The majority of fractures (68%) had an oblique fracture plane angled between 110° and 140°, with a mean angle of 123° ± 17.6°. Approximately 20% of fractures were oriented more perpendicularly (<110°), while 12% demonstrated acute angulation (>140°). Difference in fracture orientation in pars fractures have not been previously characterized but may have implications for the feasibility of surgical repair, particularly direct pars screw fixation. Fractures with steeper or more acute angles (>140°) may be less amenable to the standard intralaminar screw trajectory and may require an alternate trajectory, such as a cortical screw trajectory^20^, to maximize purchase. In addition, fractures with sharp angulation (>140°) may be better suited for lateral pars grafting, as the fracture line may not be readily accessible from a dorsal approach. However, oblique (110°-140°) and perpendicular (<110°) fractures are excellent candidates for the traditional intralaminar screw trajectory.

Pediatric pars fractures most commonly occur in the proximal third of the pars interarticularis and have a fracture orientation between 110° and 140° relative to the long axis of the pars. There is considerable variation in both pars and fracture morphology, including the total length of the pars, the width of the pars adjacent to the fracture, the fracture gap, the location of the fracture along the pars, and the fracture orientation. This variability underscores the importance of individualized planning for patient- and fracture-specific spondylolysis repairs. This study has several limitations. As a retrospective, single-center study, the findings may not be generalizable to all patient populations. In addition, race and ethnicity were not analyzed in this study which may also impact generalizability. The sample size, while moderate, limits the ability to perform more robust statistical analyses beyond descriptive statistics. In addition, as a radiographic study, correlations with symptom duration, activity level, pain scores, or clinical outcomes were not assessed. Despite these limitations, understanding the fracture location and angle can help guide optimal screw trajectory and implant development for direct spondylolysis repairs, potentially enhancing fixation strength and minimizing the risk of complications. Our study suggests that CT or MRI-based synthetic CT imaging would be useful for preoperative planning and intraoperative navigation to optimize intralaminar screw placement given the significant variability in pars fractures. Future studies incorporating clinical outcomes will be essential to determine how fracture morphology influences treatment success and long-term function.
